# High‐Pressure Modulation of Breathing Kagome Lattice: Cascade of Lifshitz Transitions and Evolution of the Electronic Structure

**DOI:** 10.1002/advs.77218

**Published:** 2026-08-24

**Authors:** Marcos Vinicius Gonçalves‐Faria, Maxim Wenzel, Yuk Tai Chan, Olga Iakutkina, Francesco Capitani, Davide Comboni, Michael Hanfland, Qi Wang, Hechang Lei, Martin Dressel, Alexander A. Tsirlin, Alexej Pashkin, Stephan Winnerl, Manfred Helm, Ece Uykur

**Affiliations:** ^1^ Helmholtz‐Zentrum Dresden‐Rossendorf Ion Beam Physics and Materials Research Dresden Dresden Germany; ^2^ Institute of Applied Physics TUD Dresden Univestity of Technology Dresden Dresden Germany; ^3^ 1. Physikalisches Institut Universität Stuttgart Stuttgart Germany; ^4^ SMIS beamline, Synchrotron SOLEIL L'Orme des Merisiers Saint‐Aubin France; ^5^ European Synchrotron Radiation Facility (ESRF) Grenoble France; ^6^ School of Physical Science and Technology ShanghaiTech University Shanghai China; ^7^ ShanghaiTech Laboratory for Topological Physics ShanghaiTech University Shanghai China; ^8^ School of Physics and Beijing Key Laboratory of Opto‐electronic Functional Materials & Micronano Devices Renmin University of China Beijing China; ^9^ Key Laboratory of Quantum State Construction and Manipulation (Ministry of Education) Renmin University of China Beijing China; ^10^ Felix Bloch Institute for Solid‐State Physics Leipzig University Leipzig Germany

**Keywords:** ${\mathrm{Fe}}_{3}{\mathrm{Sn}}_{2}$, high‐pressure, kagome metals

## Abstract

The interplay between electronic correlations, density wave orders, and magnetism gives rise to several fascinating phenomena. In recent years, kagome metals have emerged as an excellent platform for investigating these unique properties, which stem from their itinerant carriers arranged in a kagome lattice. Here, we show that electronic structure of the prototypical kagome metal, ${\mathrm{Fe}}_{3}{\mathrm{Sn}}_{2}$, can be tailored by manipulating the breathing distortion of its kagome lattice with external pressure. The breathing distortion is suppressed around 15 GPa and reversed at higher pressures. These changes lead to a series of Lifshitz transitions that we detect using broadband and transient optical spectroscopy. Remarkably, the strength of the electronic correlations and the tendency to carrier localization are enhanced as the kagome network becomes more regular, suggesting that breathing distortion can be a unique control parameter for the microscopic regime of the kagome metals and their electron dynamics.

## Graphical abstract

External pressure directly tunes the breathing distortion of the kagome lattice in Fe_3_Sn_2_, suppressing it near 15 GPa and reversing it at higher pressures. This structural modulation drives a cascade of Lifshitz transitions, reconstructs the Fermi surface, strengthens electronic correlations and carrier localization, and modifies ultrafast electron dynamics, establishing breathing distortion as a powerful control parameter for kagome‐metal electronic properties.

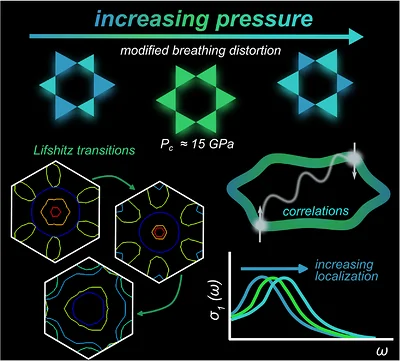

## 1 Introduction

Corner‐sharing triangular geometry of the kagome lattice combined with itinerant carriers gives rise to various exotic phenomena, such as flat‐band ferromagnetism [1, 2, 3], Wigner crystallization [4, 5], fractional/anamalous quantum Hall effect [6, 7, 8, 9], Bose–Einstein condensation [10], etc. The resulting interplay between magnetism, superconductivity, density‐wave orders, and strong electronic correlations has been investigated by various experimental methods [11, 12, 13]. Recent studies have revealed the presence of Dirac/Weyl states and flat bands [14, 15, 16, 17, 18, 19], chiral spin structures [20], and skyrmionic lattices [21, 22] in magnetic kagome metals. In contrast, non‐magnetic counterparts have shown intriguing density‐wave orders and superconductivity [23, 24, 25, 26]. Several new compounds, such as FeGe, have also been discovered that bridge the gap between these two categories [27]. Owing to the wide variety of intriguing phenomena that can be realized in itinerant kagome systems, a natural question arises about how these properties are related to the underlying kagome network and whether they can be tuned with external stimuli. External pressure is especially valuable in this context, as it allows to tune the electronic structure and band filling without introducing additional chemical disorder.

Compression of the kagome metals often affects the “observer” atoms adjacent to the kagome network, whereas the most alluring kagome d‐bands remain almost unchanged. For example, reentrant behavior of superconductivity in pressurized ${\mathrm{CsV}}_{3}{\mathrm{Sb}}_{5}$ is driven by the evolution of the Sb p‐bands of the material without affecting its kagome d‐bands [28, 29]. In this context, ${\mathrm{Fe}}_{3}{\mathrm{Sn}}_{2}$ is a rare example of the kagome lattice deformed by a breathing distortion [30] (Figure 1) that couples to the external pressure and drives pressure‐induced changes in the kagome bands, as we demonstrate in the following. At ambient pressure, ${\mathrm{Fe}}_{3}{\mathrm{Sn}}_{2}$ is a ferromagnet with a Curie temperature of 640 K [31]. It features flat bands and massive Dirac fermions [32, 33, 34, 35], a nontrivial Hall response [36, 37], intriguing non‐Fermi‐liquid behavior [38], and ultrafast spin‐wave transport [39]. Little is known about its pressure evolution, though, beyond the mere fact that lattice parameters evolve smoothly upon compression without any abrupt structural phase transitions up to at least 20 GPa [40]. On the other hand, within the $R\bar{3}m$ space group, Fe, Sn1, and Sn2 sit at the atomic positions of (x,‐x,z1), (0,0,z2), and (0,0,z3), respectively. These special Wyckoff positions suggest that the motion of the Sn1 and Sn2 atoms is confined to the out‐of‐plane direction, whereas the Fe atoms can move within all three dimensions of the kagome network under external pressure creating a pathway toward the tunability of the breathing distortion.

**FIGURE 1:**
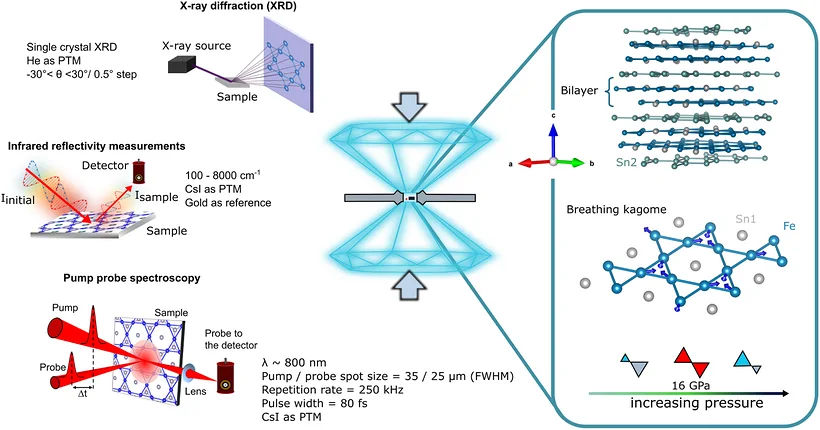
Summary of the experimental methods used in the present work: X‐ray diffraction, broadband infrared spectroscopy, and optical pump‐probe spectroscopy have been performed on single crystals prepared from the same crystal. Suitable pressure cells and pressure transmitting medium have been chosen for each method specifically. Bilayer kagome metal ${\mathrm{Fe}}_{3}{\mathrm{Sn}}_{2}$ is tuned with external pressure, leading to a gradual suppression and eventual reversal of the breathing distortion, accompanied by a cascade of Lifshitz transitions.

Here, we demonstrate the tunability of the kagome lattice of ${\mathrm{Fe}}_{3}{\mathrm{Sn}}_{2}$ via external pressure and the resulting electronic reconstruction traced by broadband and transient optical spectroscopy studies (Figure 1). Structural evolution monitored by single‐crystal x‐ray diffraction (XRD) shows the suppression of the breathing distortion around 15 GPa and its reversal at higher pressures. These changes give rise to a series of Lifshitz transitions that fully reshape the Fermi surface of ${\mathrm{Fe}}_{3}{\mathrm{Sn}}_{2}$ and change its crucial parameters, such as the correlation strength, back‐scattering of the unconventional carriers, and electronic relaxation. We show that the breathing mode of the kagome lattice is a powerful tool for tailoring the kagome d‐bands and modifying electronic properties of these materials.

## 2 Results

### 2.1 Structural Evolution of the Breathing Mode

In order to identify the structural changes, in particular the pressure evolution of the breathing mode, we performed a single crystal XRD study up to ∼ 28 GPa. No structural phase transition was observed in agreement with the previous powder XRD studies [40]. As demonstrated in Figure 2a–c, both a and c lattice parameters evolve gradually, with only a slightly more pronounced change along the c‐axis, as one might expect from a system with a layered structure like ${\mathrm{Fe}}_{3}{\mathrm{Sn}}_{2}$. Both magnetic and nonmagnetic ab initio calculations were used to estimate the compressibility of ${\mathrm{Fe}}_{3}{\mathrm{Sn}}_{2}$. Experimental pressure dependence of the unit‐cell volume is an excellent agreement with the calculation for the ferromagnetic equation of state (see Supporting Information for the fit parameters), thus indirectly suggesting that ferromagnetism is preserved up to at least 28 GPa, otherwise deviations from this equation of state would be expected.

**FIGURE 2:**
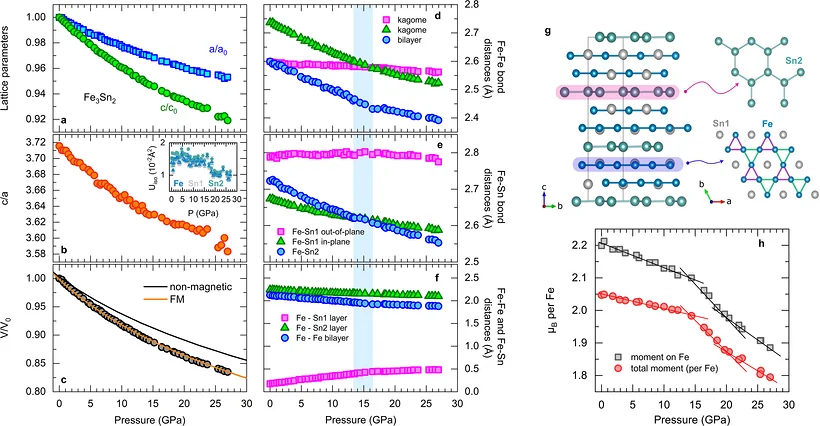
Pressure‐dependent structural parameters of ${\mathrm{Fe}}_{3}{\mathrm{Sn}}_{2}$. (a–c) Pressure‐dependent lattice parameters and the volume change. Inset in (b) shows atomic displacement parameters of Fe, Sn1, and Sn2. Overall, all the atoms show similar displacement with a slight decrease and a sudden drop at the high‐pressure range. That is expected as the system becomes denser. The black and orange lines correspond to the equations of state obtained from the DFT calculations for the nonmagnetic and ferromagnetic cases, respectively. Only the ferromagnetic calculations reproduce the experimental behavior. (d) Fe–Fe bond distance between two layers of the bilayer is gradually reduced. By contrast, the Fe‐Fe distances within the same layer of the kagome network (color code is given in **g**) cross around 15 GPa, indicating the suppression and eventual reversal of the breathing distortion. (e) Fe‐Sn bond distances. The Fe‐Sn1 (out of plane) does not change significantly, suggesting that the overall bilayer structure is robust. (f) Fe‐Fe and Fe‐Sn distances (vertical). Fe‐Sn1 shows an unexpected increase as the Sn1 layers are slightly pushed away from the kagome network. As discussed in the main text, this also has certain effects on the phonon dynamics in ${\mathrm{Fe}}_{3}{\mathrm{Sn}}_{2}$. (g) Crystal structure of ${\mathrm{Fe}}_{3}{\mathrm{Sn}}_{2}$ visualized by VESTA [68]. The breathing mode is also shown with a color code. (h) Calculated magnetic moment (total moment per Fe and moment on Fe). The total moment of 2.05 $\mu_{B}$ per Fe at ambient pressure is consistent with the literature [66]. The kink at higher pressures marks the Lifshitz transitions, as discussed in the text.

Two main features of the kagome structure in ${\mathrm{Fe}}_{3}{\mathrm{Sn}}_{2}$ are the formation of bilayers and the breathing distortion of each kagome layer therein. The main pressure effect on Sn‐atoms is that the Sn1 atom stabilizing the kagome plane is gradually pushed out of the kagome plane, whereas the Sn2 atom in between the bilayer kagome planes is gradually moved with the external pressure. On the other hand, more interesting changes occur within the kagome planes. We further track experimentally the evolution of the respective Fe–Fe distances (Figure 2d–f). The Fe–Fe bond distances decrease continuously following the lattice parameters within the bilayer structure, signaling that the coupling between these two layers is effectively increasing. The Fe–Fe distances within one kagome layer, on the other hand, show an interesting development. The Fe–Fe bonds on the consecutive corner‐sharing triangles of the kagome structure do not shrink equally; rather, the long bond decreases faster. This leads to the gradual suppression of the breathing kagome distortion until the kagome layer becomes regular at ∼15 GPa. The breathing distortion is then reversed upon further compression. These results demonstrate that the external pressure can be efficiently used to tune the kagome lattice directly. As discussed below, approaching the perfect kagome structure has significant consequences for the electronic structure of ${\mathrm{Fe}}_{3}{\mathrm{Sn}}_{2}$.

### 2.2 Pressure‐Induced Lifshitz Transitions

Next we utilized optical spectroscopy to study the electronic changes accompanying the structural evolution in ${\mathrm{Fe}}_{3}{\mathrm{Sn}}_{2}$. Previous optical studies at ambient pressure have revealed several interband transitions as well as conventional and unconventional intraband transitions in ${\mathrm{Fe}}_{3}{\mathrm{Sn}}_{2}$ [35]. All of these features can be traced under pressure. In Figure 3a,b, an exemplary decomposition of the low‐ and high‐pressure optical conductivity is given, showing a conventional Drude contribution (blue), an unconventional localization peak (green), and several interband transitions (orange) (See Supporting Information and Figure S1 for pressure dependence). Here, we applied the same decomposition method used for the other kagome metals as explained in Ref. [29].

**FIGURE 3:**
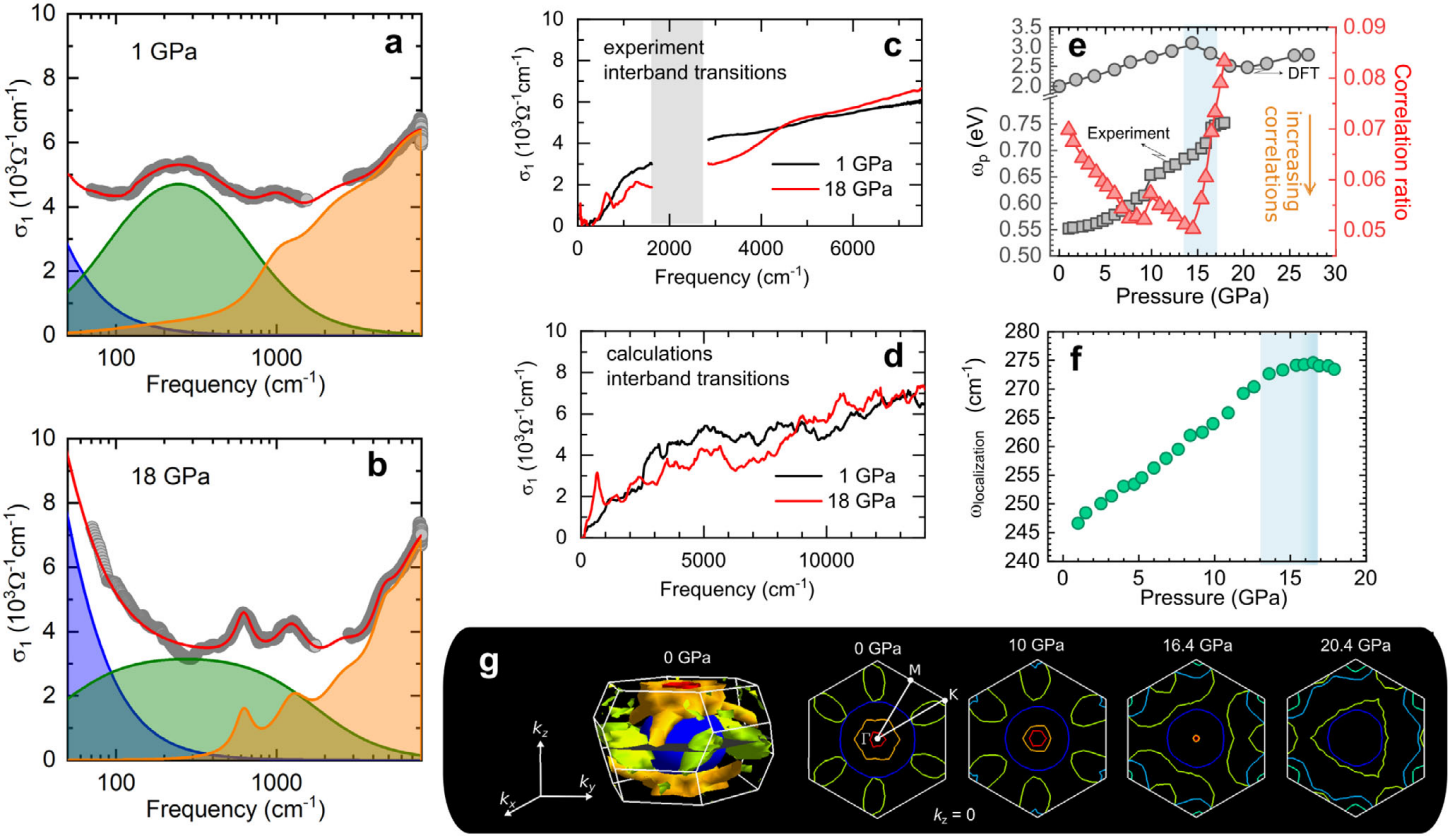
(a,b) Decomposition of the optical conductivity at the lowest and highest pressures measured at room temperature. Drude (blue), unconventional carriers (green), and the interband transitions (orange) are identified. (c,d) Comparison of the experimental and calculated interband transitions at 1‐ and 18 GPa. There are no significant changes with pressure, but subtle changes can be reproduced very well. The energy scale of the calculated optical conductivity is rescaled by ∼/2 to account for band energy renormalization in a correlated system. (e) Experimental and calculated plasma frequencies. The correlation ratio is also given on the same plot. The tendency of increasing the correlations is reversed above ∼15 GPa. (f) Pressure‐dependent resonance of the localization peak gradually increases and eventually saturates above ∼15 GPa. (g) Fermi surfaces at different pressures illustrated using FermiSurfer [69]. Chosen pressures represent the series of Lifshitz transitions.

More pronounced low‐energy interband transitions appear in the spectra with increasing pressure, while simultaneously, the MIR spectral weight is slightly suppressed. Even though the changes in the interband transitions are not very significant, they can be used to benchmark our DFT calculations. In Figure 3c,d, a comparison of the optical spectra is given for 1  and 18  GPa. The intraband transitions (namely Drude + localization) have been removed from the response to facilitate a direct comparison between the experimental interband transitions of the optical response and the DFT calculations. The suppression of the MIR spectral weight, the low energy absorptions, and the crossover energies between low‐ and high‐pressure spectra are well reproduced by the DFT calculations upon a suitable energy renormalization. This mismatch in the energy scales is not very surprising since ${\mathrm{Fe}}_{3}{\mathrm{Sn}}_{2}$ is a strongly correlated electron system [33]. However, these results indicate that the DFT calculations can be used as a guide to the changes in the electronic structure.

Intraband part of the optical conductivity gives further insights into the pressure dependence of the electronic structure. While taking into account the Drude and the localization peak, we determined the plasma frequency ($\omega_{p}^{2}\approx$ carrier density) in ${\mathrm{Fe}}_{3}{\mathrm{Sn}}_{2}$ (Figure 3e). An overall increase in the plasma frequency is observed, which is in line with the increase in the Fermi surface size. A closer look reveals non‐monotonous changes at ∼10  and 16 GPa. Sudden jumps indicate a reconstruction of the Fermi surface topology, the Lifshitz transitions [41]. Indeed, the Fermi surfaces plotted as a function of pressure (Figure 3g) reveal the appearance of new sheets at the K‐point of the Brillouin zone at these critical pressures, demonstrating the close link to the observed changes in the plasma frequency. It also indicates that the in‐plane optical conductivity might be more sensitive to the changes along the K‐point of the Fermi surface. As demonstrated below in Figure 3e, the plasma frequency calculated via DFT has a more smooth evolution possibly due to balanced changes across the zone center and zone boundary of the Fermi surface. A decrease in the former and an increasing trend in the latter is reflected as smooth changes. Furthermore, the disappearance of the Γ‐point Fermi surface has a more drastic effect on the DFT estimated plasma frequency.

Another indication of the Lifshitz transition is the kink in the pressure dependence of the magnetic moment around 15 GPa. The magnetic moment corresponds to the difference in the number of states in the spin‐up and spin‐down channels at the Fermi level, so it also reflects an abrupt change in the Fermi surface when the breathing distortion is suppressed (Figure 2h). Pressure‐dependent magnetic moment in ${\mathrm{Fe}}_{3}{\mathrm{Sn}}_{2}$ can be also accessed experimentally using ${\mathrm{Fe}}^{57}$ Mössbauer spectroscopy, but it would require low‐temperature or temperature‐dependent studies in order to disentangle the decrease in the ground‐state magnetic moment from the change in the Curie temperature with pressure. Such experiments lie beyond the scope of the present work and would be an interesting topic for future research. Further modifications of the Fermi surface are observed at higher pressures. Two Fermi surfaces at the Γ‐point disappear when the breathing distortion is reversed.

The direct comparison of the experimental plasma frequencies to those calculated from the DFT calculations can give an insight into the correlation strength in materials [42] (See Supporting Information for details). At ambient pressure, ${\mathrm{Fe}}_{3}{\mathrm{Sn}}_{2}$ stands out as one of the most correlated compounds among the different classes of kagome metals (For comparison see [42, 43]). Our pressure‐dependent study shows that ${\mathrm{Fe}}_{3}{\mathrm{Sn}}_{2}$ reaches its most correlated state with the suppression of the breathing distortion. Above this crossover, the correlation strength decreases; however, up to at least ∼ 18 GPa, it remains highly correlated. The non‐monotonic change in the correlation strength demonstrates that multiple mechanisms are at play regarding the electronic correlations in ${\mathrm{Fe}}_{3}{\mathrm{Sn}}_{2}$, and it strongly indicates the switch of the dominant contribution under pressure. The significant alteration of the Fermi surface at around 15 GPa notably affects these correlations, as well.

### 2.3 Localized Carriers

The localization peak reflects the unconventional carriers in kagome metals and is commonly observed in different members of this materials class [29, 43]. While sometimes this response can be identified as a second Drude contribution reflecting charge carriers with different scattering rates [44, 45], in most cases including ${\mathrm{Fe}}_{3}{\mathrm{Sn}}_{2}$, a displaced Drude peak due to strong localization effects is observed. These localized carriers arise from the interactions of charge carriers with low‐energy excitations, such as phonons and electric or magnetic fluctuations, leading to a back‐scattering of the electrons [46, 47]. Although different classes of kagome metals exhibit these carriers, the temperature and pressure‐dependent features depend on the details of the studied materials. However, due to the larger overlap of the electronic wavefunctions and the increased probability of the neighboring site hopping, the general expectation is that increasing pressure should delocalize these carriers, and the localization peak should shift to the lower energies, evolving into a Drude‐like contribution, and/or disappear. Indeed, such a behavior is observed upon the initial compression of the nonmagnetic kagome metal ${\mathrm{CsV}}_{3}{\mathrm{Sb}}_{5}$ [48].

In ${\mathrm{Fe}}_{3}{\mathrm{Sn}}_{2}$, on the other hand, the localization peak shifts to higher energies (Figure 3f) contrary to the expectations right before it saturates above the 15 GPa crossover. This behavior suggests that the carrier localization in the kagome metals may be related to the kagome geometry itself, because it increases as the kagome network becomes more regular upon compression. The blue shift of the localization peak can be associated with the gradual increase in the density of states (DOS) at the Fermi level. Above 10 GPa, DOS develops a peak near 0 eV reminiscent of a van Hove singularity (see Figure S6). Alternatively, assuming that localization is caused by the carrier damping by phonons, the blue shift would correspond to the phonon hardening under pressure, although no saturation around 15 GPa should be expected in this case.

### 2.4 Non‐Equilibrium Electron Dynamics

It is further instructive to address the behavior of charge carriers in the kagome metal beyond equilibrium; therefore, we expanded our investigation to ultrafast optical pump‐probe measurements under pressure. At ambient pressure, one observes coherent phonon oscillations and two distinct relaxation dynamics in this material [49]. A short relaxation time of around 1 ps was attributed to the hot electron cooling ($\tau_{1}$). A second relaxation time on the order of 10s of ps ($\tau_{2}$) was also observed, which is clearly distinct from the heat dissipation due to the lattice cooling, shown to be extremely slow (on the order of ns). The short relaxation time is expected from a metallic system such as ${\mathrm{Fe}}_{3}{\mathrm{Sn}}_{2}$. The presence of the second relaxation time in such long time scale is clearly unexpected and can be associated with the presence of unconventional, localized carriers. Two relaxation processes were also observed in other kagome metals [50, 51, 52].

Our high‐pressure optical pump‐probe study, as summarized in Figure 4 (see Supporting Information and Figures S2, S3 for the details), tracks changes in electron dynamics upon reducing the breathing distortion of the kagome lattice. With increasing pressure, $\tau_{1}$ evolves non‐monotonically. Up to around 5 GPa, the relaxation time is almost constant, whereas above 5 GPa a strong suppression is observed along with the amplitude. This sudden change in $\tau_{1}$ likely indicates the change in electron–phonon coupling as the fast relaxation process is governed by the electron–phonon coupling strength. $\tau_{2}$, on the other hand, increases significantly with increasing pressure without any pronounced anomalies. With increasing pressure, both relaxation processes approach the values observed in the closely related $Re{\mathrm{Mn}}_{6}{\mathrm{Sn}}_{6}$ systems [53]. These compounds possess well separated, non‐distorted kagome layers. The fact that the relaxation dynamics in ${\mathrm{Fe}}_{3}{\mathrm{Sn}}_{2}$ approach those of $Re{\mathrm{Mn}}_{6}{\mathrm{Sn}}_{6}$ within the pressure range where the breathing mode is suppressed is particularly interesting and indicates the universal behavior of the electron dynamics in the kagome metals manifesting an intricate interplay between the kagome network and the relaxation dynamics.

**FIGURE 4:**
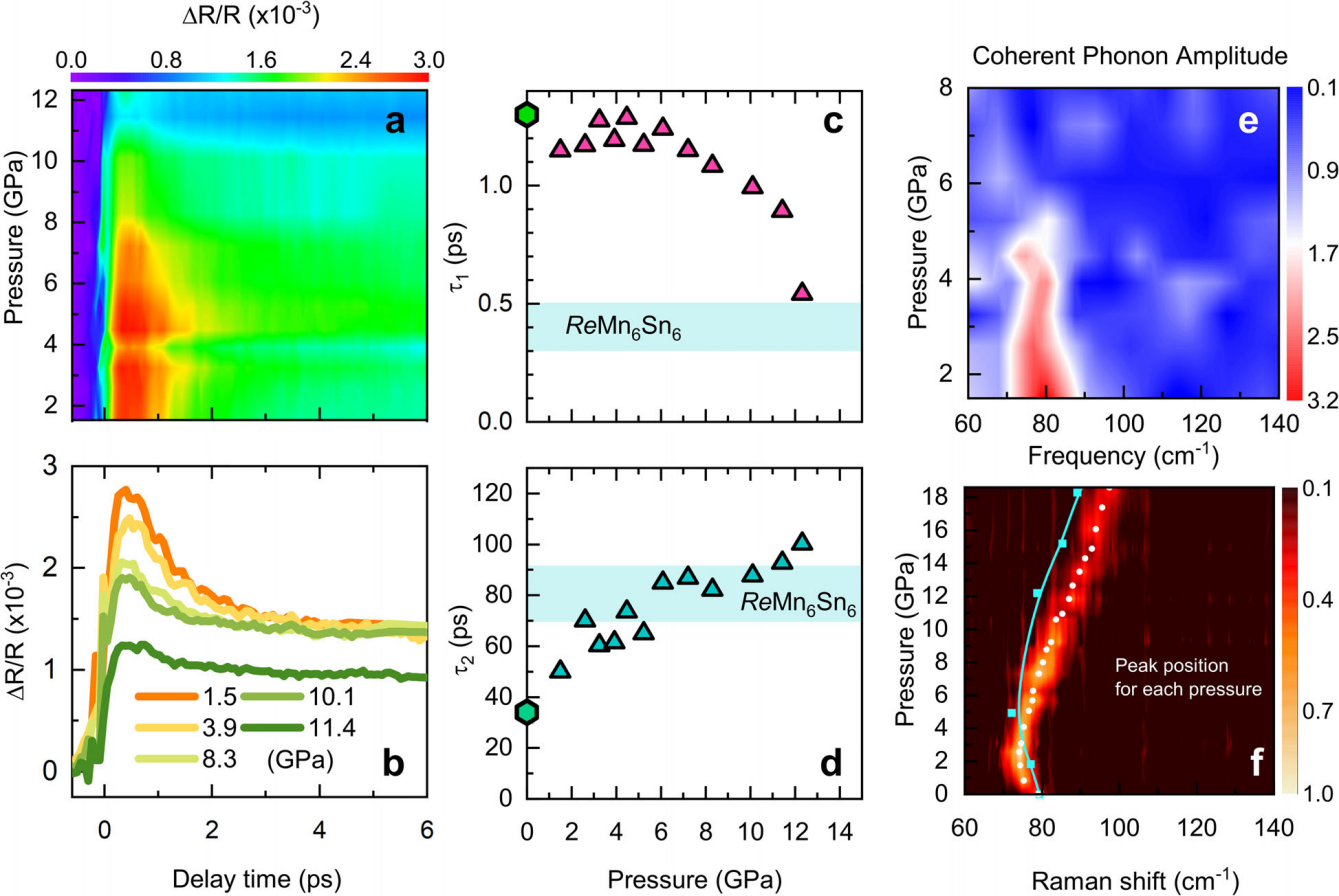
(a,b)Pressure‐dependent transient reflectivity. (c,d) Relaxation parameters are obtained via the exponential fit of the transient reflectivity (see Supporting Information for the details), where $\tau_{1}$ and $\tau_{2}$ represent the conventional and unconventional carriers, respectively. Ambient pressure data is from Ref. [49] (e) Pressure‐dependent coherent phonon amplitude. A 2.4 THz phonon is identified in the transient reflectivity and disappears above ∼4‐5 GPa. (f) Pressure‐dependent Raman shift of the 2.4 THz $A_{1g}$ phonon. A phonon softening up to ∼4 GPa is observed, followed by the gradual hardening of the mode. White points are the resonance frequency of the mode obtained with a Lorentzian fit (see Supporting Information for details). Blue points are the calculated phonon frequency via DFT. A softening also observed in DFT suggests the lattice tendency for the softening up to around 5 GPa.

External pressure has a significant impact on coherent phonon oscillations, as well. Robust phonon oscillations at ambient pressure are ascribed to the 2.4 THz $A_{1g}$ mode with the out‐of‐plane displacements of the Sn1 atoms that center the hexagons of the kagome network. It is presently unclear why this mode is strongly coupled to the electronic background, even though it does not change the positions of Fe atoms. However, the Fe atoms may be affected indirectly, because Sn1 stabilizes the kagome network.

With increasing pressure, the coherent phonon oscillations weaken and gradually disappear above around 5 GPa (Figure 4e). Interestingly, this pressure also corresponds to the pronounced suppression of the fast relaxation process, further evidencing the unconventional interplay between electrons and phonons in ${\mathrm{Fe}}_{3}{\mathrm{Sn}}_{2}$. While the energy resolution of our pump‐probe experiments is not good enough to resolve the exact frequency dependence of this mode, a Raman study (details are given in Supporting Information and Figure S4) clearly shows the non‐monotonic behavior (Figure 4f). The 2.4 THz $A_{1g}$ Sn1‐mode softens with increasing pressure up to around 4–5 GPa, followed by the expected phonon hardening at higher pressures. This behavior is well reproduced by our DFT calculations (Figure 4f, blue dots).

It can be ascribed to the two competing effects. On the one hand, pressure shifts Sn1 out of the Fe kagome plane and makes the out‐of‐plane displacements easier. On the other hand, compression along c increases the frequencies of the out‐of‐plane modes. Therefore, the behavior of the $A_{1g}$ mode itself is not anomalous, but its coupling to the electronic background is apparently reduced as the Sn1 atoms move out of the kagome plane under pressure. This observation reinforces the scenario of ${\mathrm{Fe}}_{3}{\mathrm{Sn}}_{2}$ as the kagome metal with predominantly d‐electrons of the kagome layer forming states near the Fermi level, in contrast to ${\mathrm{CsV}}_{3}{\mathrm{Sb}}_{5}$ and many other kagome metals where p‐states of non‐kagome atoms also play a significant role and underlie pressure evolution of the electronic properties [54].

## 3 Conclusion

Compression of ${\mathrm{Fe}}_{3}{\mathrm{Sn}}_{2}$ has a direct impact on its electronic structure by modifying the breathing distortion of the kagome lattice. Increasing pressure leads to the suppression of the breathing distortion around 15 GPa and to its eventual reversal at higher pressures. This fundamental change in the crystal structure causes a cascade of Lifshitz transitions evidenced by several experimental signatures: (1) anomaly in pressure dependence of the magnetic moment; (2) non‐monotonic changes in the plasma frequency and correlation strength; (3) enhancement of the localized carriers; (4) non‐monotonic changes in the relaxation dynamics and transient reflectivity. Using DFT calculations benchmarked by a direct comparison to the experimental optical conductivity, we identify two Lifshitz transitions with the appearance of a new Fermi surface sheet near K around 10 GPa and the disappearance of the sheets near Γ, along with the merge of the pockets near the zone boundary, around 15 GPa. The suppression of the breathing distortion fully re‐shapes the Fermi surface and strongly modifies electron dynamics in the kagome metal. Another reconstruction of the Fermi surface is expected at even higher pressures where the breathing distortion is reversed. This regime may be particularly unusual, because it is characterized by the same correlation strength as at ambient pressure, but with the substantially increased carrier concentration.

Thanks to its layered crystal structure, ${\mathrm{Fe}}_{3}{\mathrm{Sn}}_{2}$ shows anisotropic compressibility and the reduction in c/a under pressure, similar to other kagome metals. The unique features of this compound are the absence of non‐kagome p‐states near the Fermi level and the breathing distortion of the Fe layers that can be changed and even reversed under pressure. This combination of structural and electronic properties renders hydrostatic pressure a direct control channel for modifying electronic structure of a kagome metal.

## 4 Experimental Section

### 4.1 Single Crystal X‐ray Diffraction Measurements

The structural study was performed on a single crystal of ${\mathrm{Fe}}_{3}{\mathrm{Sn}}_{2}$. A diamond anvil cell (DAC) with 500 μm culet size was used in the measurements. The pressure‐transmitting medium was helium to ensure the high hydrostaticity in the pressure cell. Pressure values were calibrated using the ruby luminescence technique [55]. Single‐crystal X‐ray diffraction (XRD) measurements were performed at room temperature in the ID15b beamline of ESRF. The sample‐to‐detector distance was calibrated using a Si standard and a vanadinite (${\mathrm{Pb}}_{3}$(${\mathrm{VO}}_{4}$)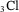) single crystal; the distance‐to‐detector was approximately 180 mm. Diffraction data were collected with the x‐ray wavelength of 0.4099 Å (convergent monochromatic beam with E ≈ 30 keV and ∼200 mA) with 0.5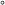 cell rotations about the ϕ axis. The data were integrated using Crysalis Pro [56]. About 500 reflections with 130 unique reflections were used to refine the isotropic atomic displacement parameters and the atomic coordinates of Fe, Sn1, and Sn2 in the $R\bar{3}m$ symmetry. The refinements were performed in JANA2006 [57] against the data collected at pressures up to 28 GPa. Further details of the structure refinements can be found in the supplementary materials (see Tables S1, S2 and Figure S5) and in the associated datasets [58, 59].

### 4.2 Infrared Reflectivity Measurements

For the optical measurements, a sample with dimensions of 150μm× 150μm× 50μm was prepared from the as‐grown single crystal. An as‐grown surface is used for the experiments. High‐pressure reflectivity measurements were performed at room temperature at the SMIS beamline of the SOLEIL synchrotron, France, on a homemade horizontal microscope with custom Schwarzschild objectives (NA = 0.5). A diamond anvil cell (DAC) with type‐IIa diamond anvils and a culet of 600μm diameter was utilized. CsI powder is used as the pressure‐transmitting medium, ensuring a good diamond‐sample interface [60]. The sample and Ruby spheres used as pressure gauges were placed inside a stainless steel gasket with a 200μm diameter hole. The pressure was determined by monitoring the calibrated shift of the ruby R1 fluorescence line as described in Ref. [55]. The reflectivity spectra at the sample‐diamond interface were recorded in a broad spectral range of 150–10000 ${\mathrm{cm}}^{-1}$ by a Thermo‐Fisher iS50 interferometer with KBr and solid substrate beamsplitters, using an MCT detector and a liquid helium‐cooled bolometer. The reflectivity of a gold foil loaded into the DAC at ambient pressure served as a reference. Other optical quantities were obtained using standard Kramers–Kronig (KK) analysis considering the sample‐diamond interface as explained in SM.

### 4.3 Ultrafast Optical Pump‐Probe and Raman Measurements

Pressure‐dependent transient reflectivity and Raman spectroscopy were measured using the same diamond anvil cell (DAC). In both cases, small pieces of ${\mathrm{Fe}}_{3}{\mathrm{Sn}}_{2}$ (around 150–200 μm diameter and 30–40 μm thickness) single crystals were placed inside an Almax Plate DAC (49 mm diamond anvil diameter and 10.5 mm working distance to the sample). The pressure inside the DAC was monitored using the standard ruby fluorescence [55]. CsI powder was used as the pressure‐transmitting medium for the time‐resolved experiments to ensure direct contact between the sample surface and the diamond. For Raman measurements, silicon oil was the pressure‐transmitting medium.

- **Optical pump‐probe**: High‐pressure optical pump‐probe experiments up to 12 GPa were performed using the reflection geometry. For the pump and probe, we used laser pulses centered at λ = 800 nm, with a pulse duration of around 80 fs. The setup is based on a Ti: sapphire laser amplifier operating with a 250 kHz repetition rate. Pump and probe spots were focused on the sample surface inside the DAC and were around 35 and 25 μm (FWHM) diameter, respectively. Pump‐probe traces were measured up to 130 ps, with a finer time steps during the first 8 ps to resolve the coherent phonon.
- **Raman spectroscopy**: Raman experiments up to 18 GPa were performed with a HORIBA commercial system. A 532 nm laser was used for the incoming beam to excite the compound. The laser is focused on the sample surface with a 50x magnification objective, and the spot size was around 5 μm diameter. The contributions from the diamond (starting around 160 ${\mathrm{cm}}^{-1}$) and the elastic scattering at lower wavenumbers were subtracted from the original data to focus on the $A_{1g}$ phonon.

### 4.4 Calculations Details

Full‐relativistic DFT calculations were performed in the FPLO (magnetic moments, density of states) [61] and Wien2K (Fermi surface and optical response) [62, 63] and VASP (equation of state, phonons) [64] codes using Perdew–Burke–Ernzerhof (PBE) exchange‐correlation potential [65] and the 24×24×24k‐mesh that ensures convergence of the Fermi energy. Lattice parameters and atomic positions were fixed to their experimental values determined from our XRD experiments. Spin‐orbit coupling was included in the calculations, and the ferromagnetic order of the sample has been taken into account by assuming the spins aligned along the c‐axis, in line with the experimental magnetic structure at ambient pressure and room temperature. The obtained total magnetic moment per Fe at ambient pressure is ∼2.05 $\mu_{B}$, consistent with the experimental results [66]. Optical conductivity was calculated using the optic module [67] on the denser k‐mesh with up to 50×50×50 points. Frequencies of Γ‐point phonons were obtained from the built‐in procedure with frozen atomic displacements of 0.015 Å.

## Conflicts of Interest

The authors declare no conflicts of interest.

## Supporting information


**Supporting File**: advs77218‐sup‐0001‐SuppMat.pdf.

## Data Availability

The data that support the findings of this study are openly available in RODARE at https://doi.org/10.14278/rodare.4804.
