# Supplementary material for: High‐Pressure Modulation of Breathing Kagome Lattice: Cascade of Lifshitz Transitions and Evolution of the Electronic Structure
Source: Adv Sci (Weinh). 2026 Aug 24:e77218. Online ahead of print. doi: 10.1002/advs.77218 (PMC13502279; doi:10.1002/advs.77218)
Supplement: Supplementary file 1 [file ADVS-9999-e77218-s001.pdf]

Supporting Information

High-Pressure Modulation of Breathing Kagome Lattice: Cascade of Lifshitz Transitions and Evolution of the Electronic Structure

Marcos V. Goncalves-Faria, Maxim Wenzel, Yuk T. Chan, Olga Iakutkina, Francesco Capitani, Davide Comboni, Michael Hanfland, Qi Wang, Hechang Lei, Martin Dressel, Alexander A. Tsirlin, Alexej Paskin, Stephan Winnerl, Manfred Helm, Ece Uykur[*]

*Corresponding author email: e.uykur@hzdr.de

Supplementary information contents:

1. Supplementary text
    1.1. Optical variables under pressure
    1.2. Electronic correlations
    1.3. Details of pump-probe measurements and analysis
    1.4. High-pressure Raman measurements
    1.5. Structure refinement parameters and equation of state
2. References
3. Tables with data collection and refined structural parameters at 1 and 26 GPa (Table S1 and S2)
4. Supplementary figures S1-S6

1 - Supplementary Text

1.1 - Optical variables under pressure

In our high-pressure infrared spectroscopy measurements, the reflectivity spectrum of Fe_3Sn_2, $R_{sd}(\omega)$ is measured at the sample-diamond interface in the diamond anvil cell (DAC) which is expressed as

$$R_{sd}(\omega) = R_{gd}(\omega)\frac{I_{sd}(\omega)}{I_{gd}(\omega)} \quad (1)$$

Here, $I_{sd}(\omega)$ is the reflected light intensity at the sample-diamond interface, and $I_{gd}(\omega)$ is at the gold-diamond interface. At each pressure, the reflectivity of the sample has been corrected for the gold foil reflection with the $R_{gd}(\omega)$. Thus, the above equation gives us the absolute reflectivity $R_{sd}(\omega)$ obtained at the sample-diamond interface.

During the Kramers-Kronig (KK) analysis of the reflectivity at the sample-diamond interface, the standard relation between the phase and the reflectivity needs to be corrected, which gives an additional term (second term in the right-hand side of Eq. 2). Namely, the presence of a medium with the refractive index larger than 1 (in the case of diamond it is 2.38) brings an extra phase
shift.

$$\theta(\omega_0) = -\frac{\omega_0}{\pi} P \int_0^{+\infty} \frac{\ln R_{sd}(\omega)}{\omega^2-\omega_0^2} d\omega + \left[\pi - 2arctan\frac{\omega_\beta}{\omega_0}\right] \quad (2)$$

P is the principal value of the complex response function. ω_β is the position of the reflectivity pole on the imaginary axis of the complex plane. For the case of measurements performed at the sample-vacuum interface, ω_β goes to infinity, eliminating the second term and leading to the standard KK analysis.

ω_β is a priori an unknown parameter; however, it can be estimated by comparing the lowest pressure optical conductivity (in our case, 1 GPa spectrum is used) with the ambient-pressure one, which can be obtained through the regular KK analysis. We found the best fitting for the parameter $\omega_\beta \approx$ 25.000 cm^{-1}.

In Fig. S1, pressure-dependent reflectivity and the obtained optical conductivity are given.

1.2 - Electronic correlations

The strength of the electronic correlations can be assessed by the comparison of the plasma frequencies obtained via experiment and DFT with the following equation:

$$correlation\ ratio = \frac{\omega_p^2\ (experiment)}{\omega_p^2\ (DFT)} \tag{3}$$

The experimental plasma frequency can be calculated using the spectral weight (SW) of the intraband transitions, which includes contributions from both Drude and localization. In this study, we modeled our optical conductivity considering Drude, localization, and interband transitions. After subtracting the interband transitions, we determined the total spectral weight of the intraband transitions using the following equation:

$$\omega_p^2 = SW = \frac{120}{\pi}\int_0^{\omega_c} \sigma_1(\omega) d\omega \tag{4}$$

Here, ω_c is chosen as the upper limit of the measurement window, which is enough to cover the contributions from Drude and localization.

1.3 - Details of pump-probe measurements and analysis

Figure S2(a) shows the transient reflectivity data at 1.5 GPa until 60 ps. The equation used to fit the traces is also shown in this panel. The same function was used for all pressure points, which has also been used before for Fe_3Sn_2 outside the pressure cell. Panels (b) and (c) from Figure S2 show the pressure evolution of the fitting parameters C1 and C2, respectively. C1 corresponds to the amplitude at time zero of the faster relaxation process (green line in panel (a)). It is attributed to the relaxation of hot electrons through electron-phonon interactions, which is the expected metallic behavior for this experiment. Interestingly, C1 reduces drastically with increasing pressure, indicating the excitation of fewer charge carriers at higher pressures. C2, on the other hand, shows a more constant behavior as a function of pressure. The origin of this longer relaxation process (blue line in panel (a)) is still not fully understood, despite being present in many of the kagome metals that have been measured with similar experiments *(1-3)*.

Coherent phonon is obtained following the procedure described in Figure S3. The double exponential fit of the transient reflectivity is subtracted from the pump-probe trace. The frequency of the phonon mode can be obtained via the Fourier transform of the remaining oscillatory signal. As seen from Figure S3(b), the amplitude of the phonon mode is gradually suppressed and disappears above ~5 GPa within the noise level of our experiments.

1.4 - High-pressure Raman measurements

In order to follow the phonon behavior at higher pressures and compare it with the optical pump probe results presented in the main text, we performed high-pressure Raman spectroscopy. The white points in Figure 4(f) in the main text represent the peak position obtained after fitting the Raman data, as shown in Figure S4(a). The same fitting procedure was used for all the pressure points.

1.5 - Structure refinement parameters and equation of state

Fits of the energy-vs-volume curves with the Murnaghan equation of state *(4, 5)* return the equilibrium volume V_0 = 489.379(2) Å^3/f.u., bulk modulus B_0 = 92.78(9) GPa, and pressure derivative of the bulk modulus B_0' = 4.75(8).

In Figure S5(a-c), the observed and calculated reflection intensities for the selected pressures are compared. The refinement residuals as a function of pressure are also depicted in Fig. S5(d). The R_{int} values show the quality of averaging over equivalent reflections, the values of R_{obs} illustrate the match between the observed and calculated intensities (quality of the structure refinement). Both *R*-values remain quite low across the whole pressure range of our study, indicating not only high quality of the XRD data, but also the high accuracy of the structural parameters obtained in the refinement.

The displacement parameters of all atoms were refined isotropically. We also attempted to refine ADP’s anisotropically, but this led to non-positively defined tensors at some of the pressures.

References:

(1) M. Tuniz, A. Consiglio, D. Puntel, C. Bigi, S. Enzner, G. Pokharel, P. Orgiani, W. Bronsch, F. Parmigiani, V. Polewczyk, P. D. C. King, J. W. Wells, I. Zeljkovic, P. Carrara, G. Rossi, J. Fujii, I Vobornik, S. D. Wilson, R. Thomale, T. Wehling, G. Sangiovanni, G. Panaccione, F. Cilento, D. Di Sante, F. Mazzola, Dynamics and resilience of the unconventional charge density wave in ScV_6Sn_6 bilayer kagome metal. *Commun. Mater*. **4**, 103 (2023).

(2) Z. X. Wang, Q. Wu, Q. W. Yin, C. S. Gong, Z. J. Tu, T. Lin, Q. M. Liu, L. Y. Shi, S. J. Zhang , D. Wu, H. C. Lei, T. Dong, and N. L. Wang, Unconventional charge density wave and photoinduced lattice symmetry change in the kagome metal CsV_3Sb_5 probed by time-resolved spectroscopy. *Phys. Rev. B* **104**, 165110 (2021).

(3) Y. Liu, Y. Han, J. Yu, H. Zhang, Q. Yin, H. Lei, J. Hu, D. Zhang, Visualizing electron–phonon and anharmonic phonon–phonon coupling in the kagome ferrimagnet $GdMn_6Sn_6$. *Applied Physics Letters* **122**, 251901 (2023).

(4) Birch, F. Finite elastic strain of cubic crystals. *Phys. Rev*. **71**, 809–824 (1947).

(5) Holzapfel, W. Equations of state for solids under strong compression. *Zeitschrift Für Kristallographie - Crystalline Materials* **216**, 473–488 (2001).

Table S1: Details of data collection and refined structural parameters for Fe_3Sn_2 at 1 GPa.

$a = b = 5.3194(3)$ Å, $c = 19.734(13)$ Å

$V = 483.6(3)$ Å^3

$R\bar{3}m$

$\lambda = 0.40990$ Å

$2\theta_{\rm min} = 2.62°$, $2\theta_{\rm max} = 21.43°$

$-9 \leq h \leq 9$, $-8 \leq k \leq 8$, $-11 \leq l \leq 8$

Number of total/unique reflections = $508/132$

$R_{int} = 0.0171$, $R_{I>3\sigma(I)} = 0.028$, $wR_{I>3\sigma(I)} = 0.0353$

Atom	x/a	y/b	z/c	$U_{\rm iso}$ (Å^2)
Fe	0.49581(9)	0.50418(3)	0.1131(1)	0.0114(3)
Sn1	0	0	0.1034(1)	0.0127(3)
Sn2	0	0	0.3316(1)	0.0151(3)

Table S2: Details of data collection and refined structural parameters for Fe_3Sn_2 at 26 GPa.

$a = b = 5.0858(10)$ Å, $c = 18.22(4)$ Å

$V = 408.2(9)$ Å^3

$R\bar{3}m$

$\lambda = 0.40990$ Å

$2\theta_{\rm min} = 2.74°$, $2\theta_{\rm max} = 20.31°$

$-8 \leq h \leq 8$, $-8 \leq k \leq 8$, $-8 \leq l \leq 9$

Number of total/unique reflections = $421/102$

$R_{int} = 0.0253$, $R_{I>3\sigma(I)} = 0.049$, $wR_{I>3\sigma(I)} = 0.056$

Atom	x/a	y/b	z/c	$U_{\rm iso}$ (Å^2)
Fe	0.5011(2)	0.5040(3)	0.1149(3)	0.0086(6)
Sn1	0	0	0.0884(3)	0.0089(5)
Sn2	0	0	0.3338(3)	0.0098(5)

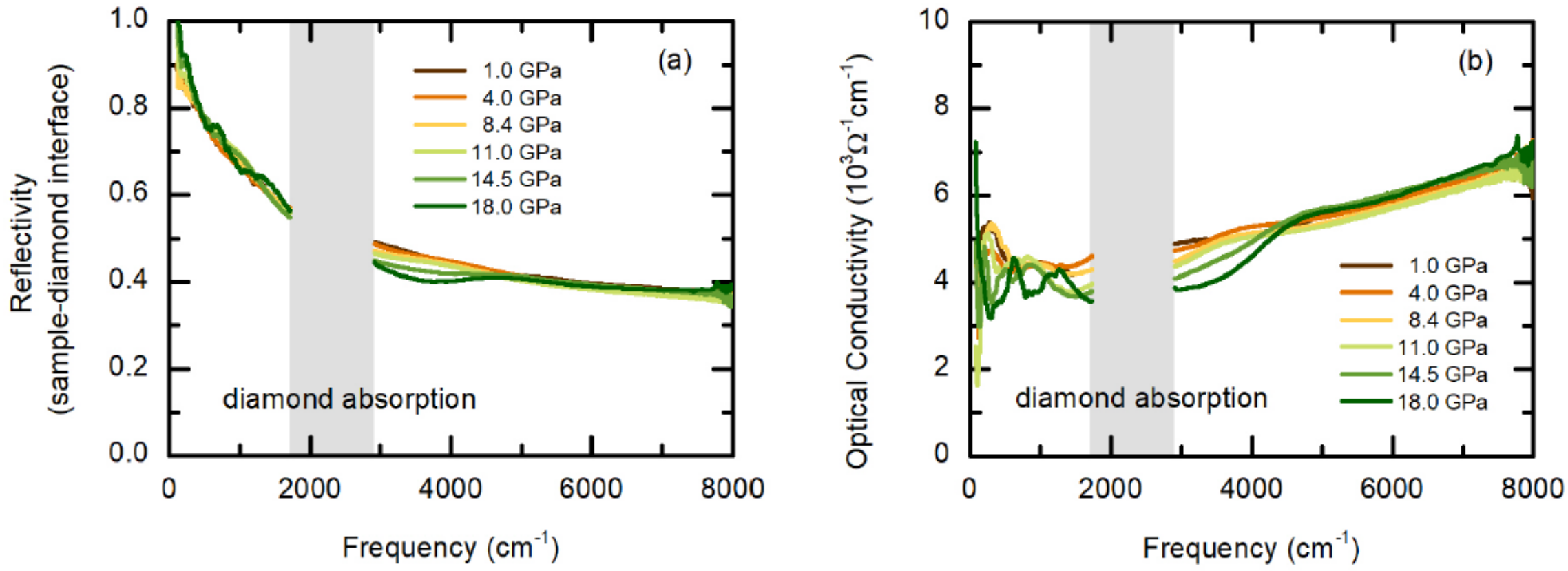

Fig. S1.
(a) Pressure-dependent reflectivity and (b) optical conductivity. Shaded areas are diamond absorptions.

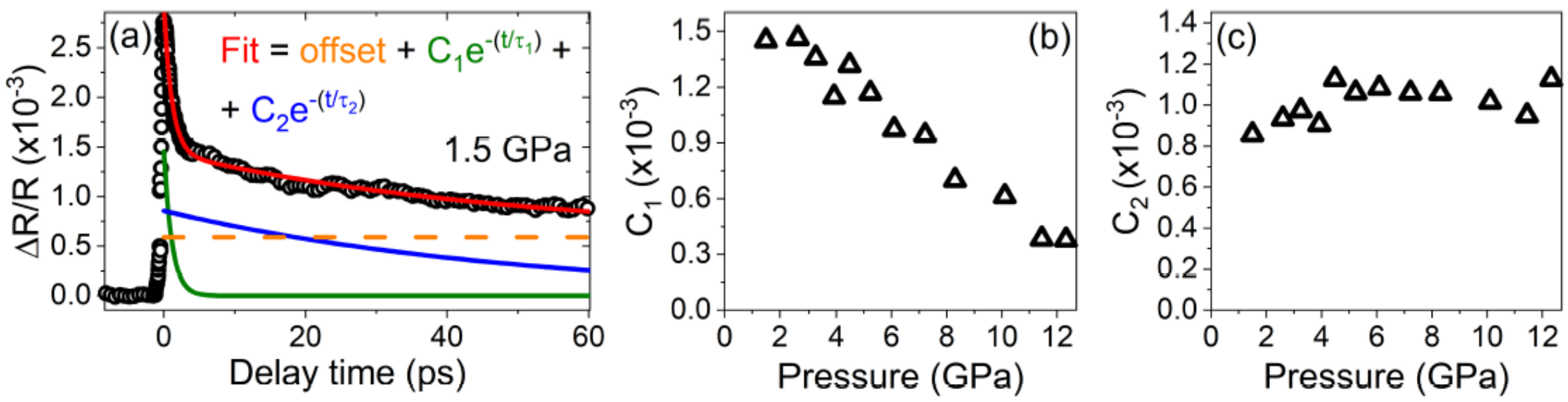

Fig. S2.
(a) Fit procedure of the transient reflectivity spectra. The relaxation times are given in the main text Figure 4(c,d). (b,c) The amplitude of the different relaxation times as described in the equation given in (a).

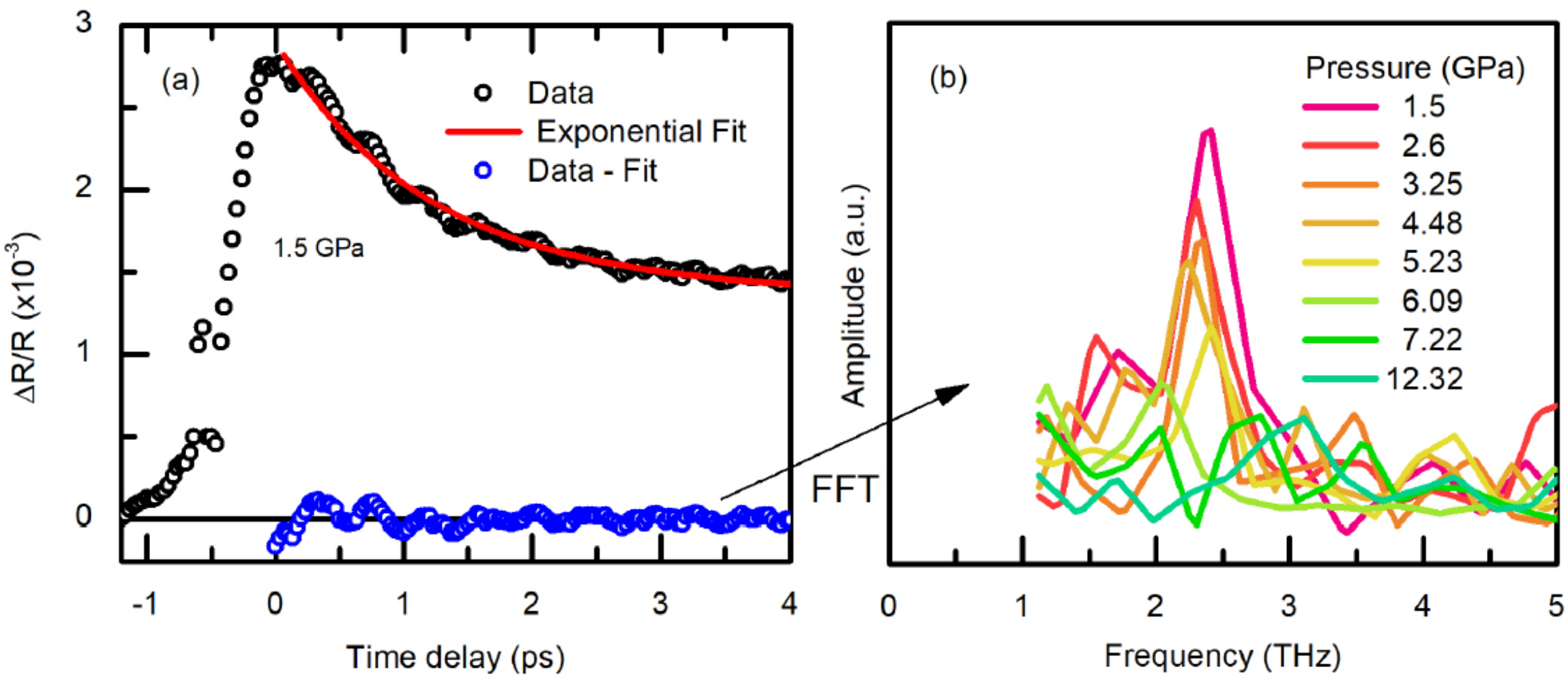

Fig. S3.
(a) Obtaining the coherent phonon signal. (b) The amplitude of the Fourier transform of the oscillatory signal signifying the phonon mode that disappears above ~5 GPa.

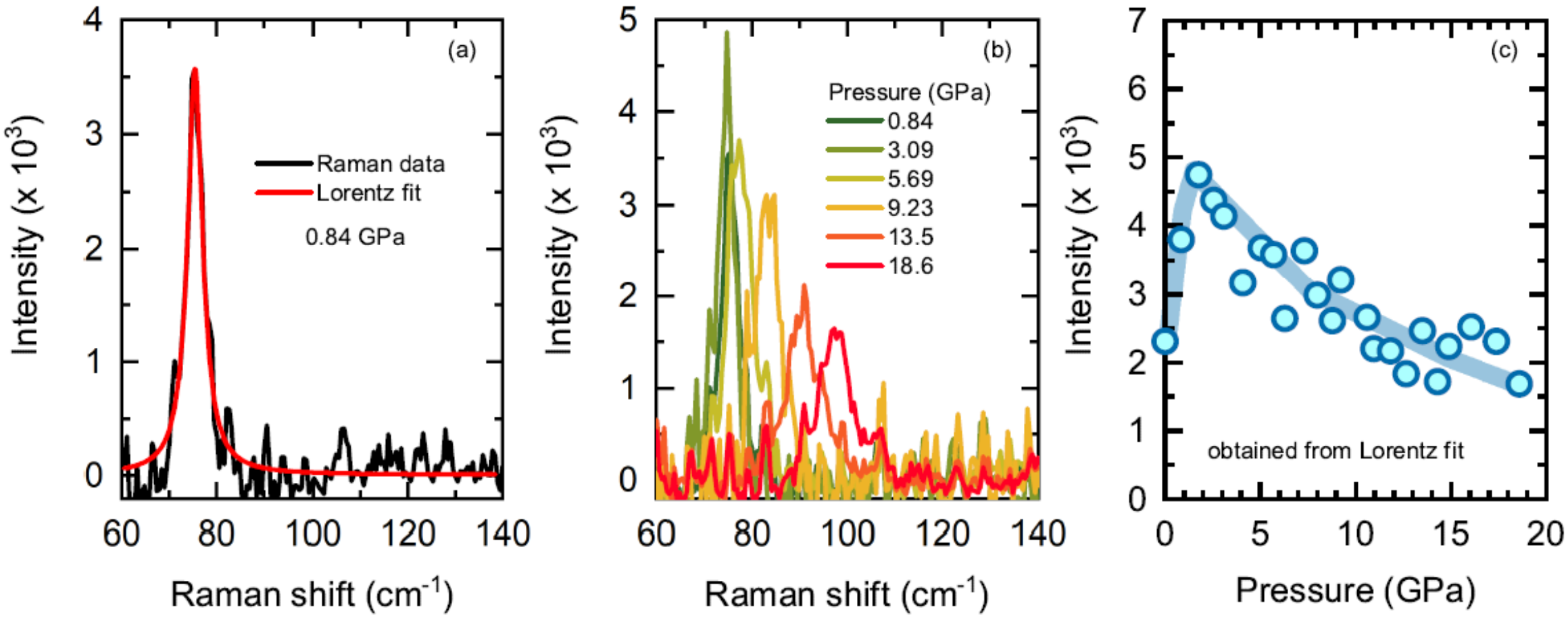

Fig. S4.
(a) Lorentzian fit of the Raman data. The same fitting was used for all the pressure points. (b) Pressure evolution of the A_{1g} mode. (c) The pressure-dependent intensity of the phonon mode obtained by the Lorentzian fit.

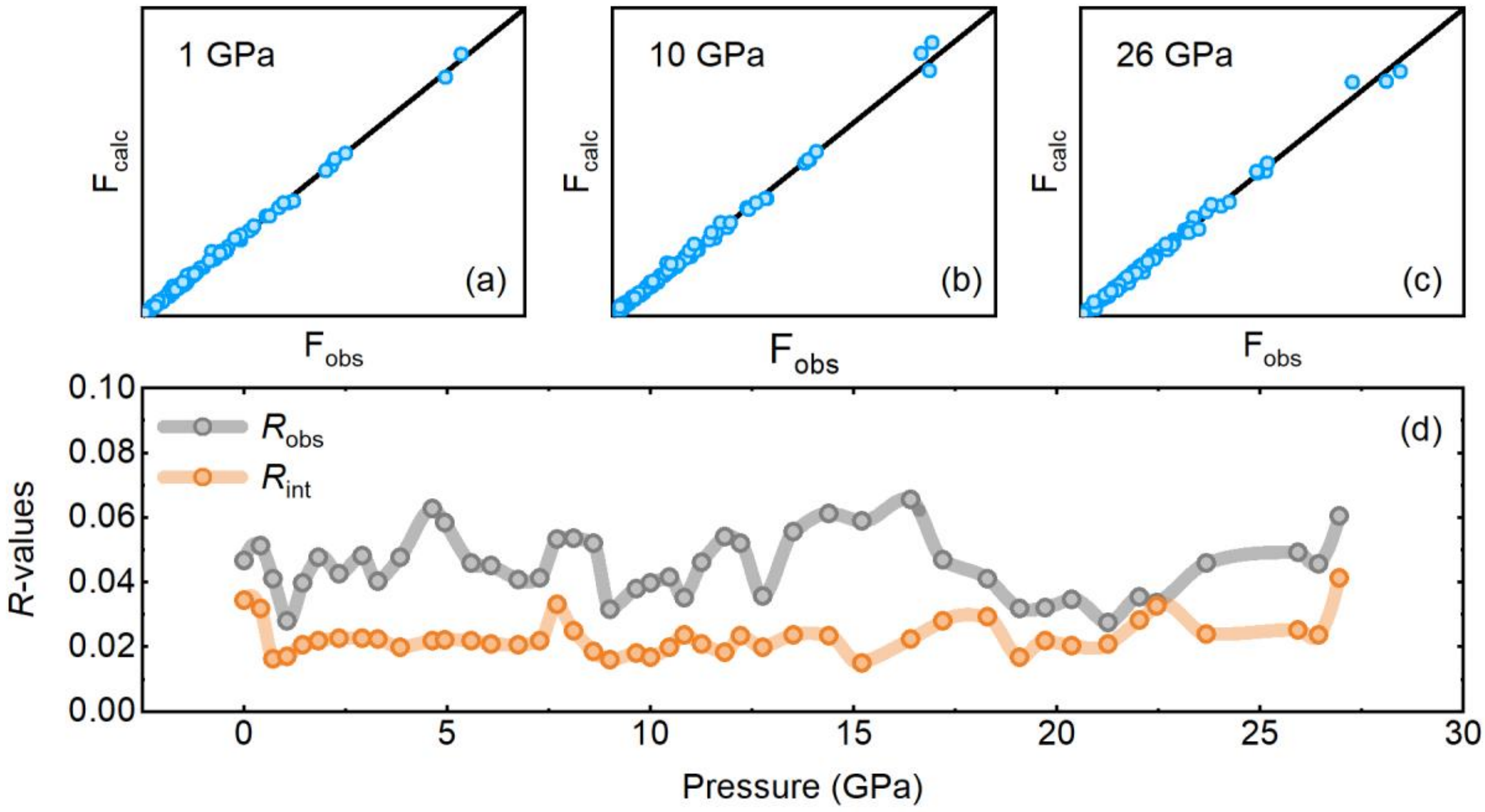

Fig. S5.
(a-c) Comparison between the observed and calculated reflection intensities at 1 GPa, 10 GPa, and 26 GPa. (d) The refinement residuals as a function of pressure. The R_{int} values show the quality of averaging over equivalent reflections, the values of R_{obs} illustrate the match between the observed and calculated intensities (quality of the structure refinement).

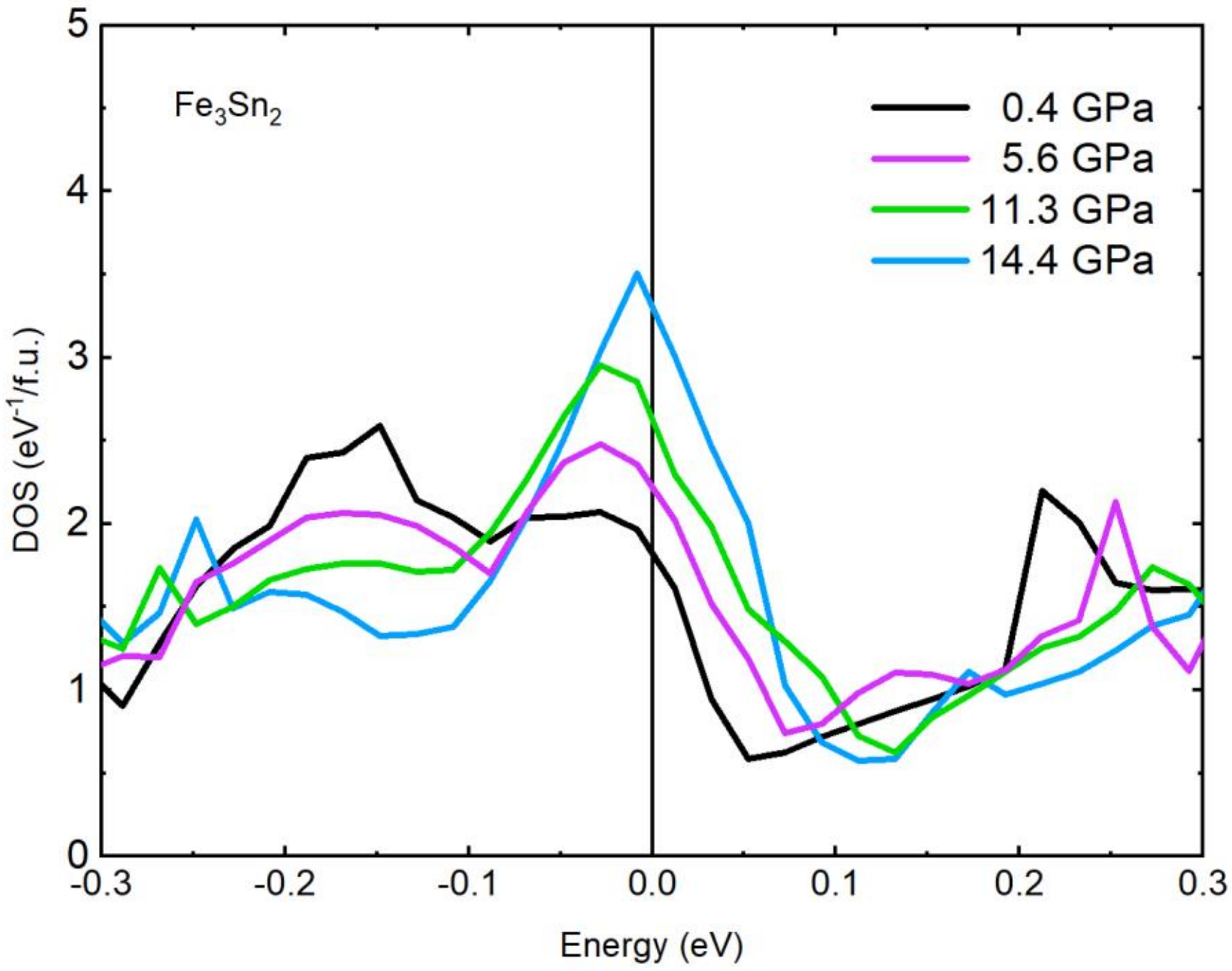

Fig. S6.

Pressure evolution of density of states (DOS) around Fermi energy. A gradual increase at the Fermi energy is visible.
